# Microcatheter-facilitated alcohol septal ablation for residual left ventricular outflow tract obstruction

**DOI:** 10.1093/ehjcr/ytae485

**Published:** 2024-09-10

**Authors:** Takashi Hiruma, Mitsunobu Kitamura, Itaru Takamisawa, Morimasa Takayama

**Affiliations:** Hypertrophic Cardiomyopathy Center, Department of Cardiology, Sakakibara Heart Institute, 3-16-1 Asahi-cho, Fuchu, Tokyo 183-0003, Japan; Hypertrophic Cardiomyopathy Center, Department of Cardiology, Sakakibara Heart Institute, 3-16-1 Asahi-cho, Fuchu, Tokyo 183-0003, Japan; Hypertrophic Cardiomyopathy Center, Department of Cardiology, Sakakibara Heart Institute, 3-16-1 Asahi-cho, Fuchu, Tokyo 183-0003, Japan; Hypertrophic Cardiomyopathy Center, Department of Cardiology, Sakakibara Heart Institute, 3-16-1 Asahi-cho, Fuchu, Tokyo 183-0003, Japan

**Figure:**
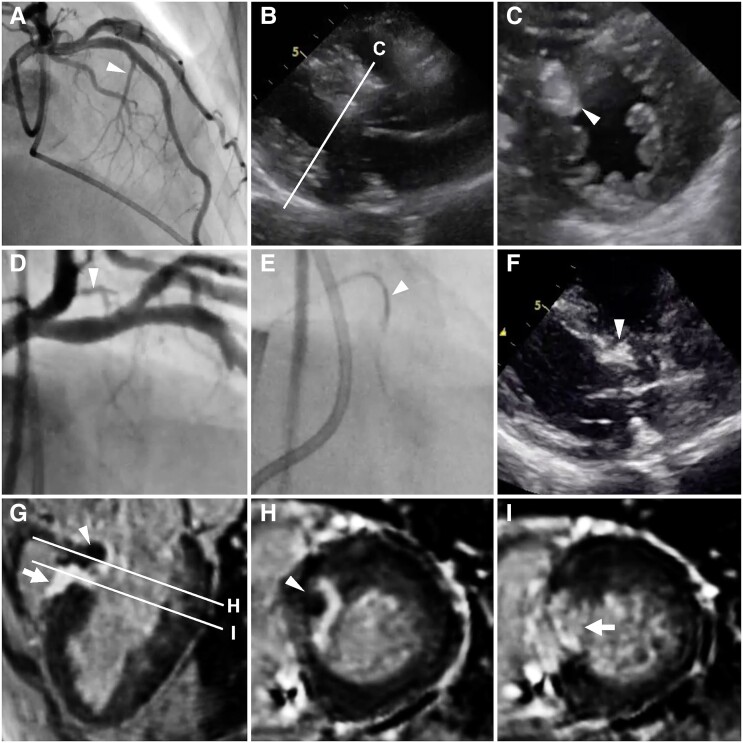


A 65-year-old female patient with obstructive hypertrophic cardiomyopathy was referred to our hospital for septal reduction therapy. Echocardiography revealed left ventricular outflow tract obstruction (LVOTO) with systolic anterior motion of the mitral leaflet and no abnormal structure related to the obstruction. Coronary angiography identified the major septal branch originating from the left anterior descending artery to be a target branch for alcohol septal ablation (ASA) (*Panel A*). In the initial procedure, under septal branch occlusion by a 2 mm over-the-wire balloon (see *Movie S1*), the culprit myocardial bulge was ablated with 2 mL of ethanol (*Panels B* and *C*), with the resting gradient reduced from 100 to 3 mmHg and peak serum creatine kinase level of 1330 U/L. One year later, exertional dyspnoea recurred with LVOTO at the most-basal septum. In coronary angiography, a sub-branch from the left circumflex artery was deemed to be a target vessel for ASA (*Panel D*). In the repeat procedure, we used a Corsair Pro microcatheter [tip: 1.3 Fr (0.42 mm), shaft: 2.6 Fr (0.87 mm), Asahi Intec, Nagoya, Japan]. A tip-injection into the microcatheter wedged on the sub-branch confirmed the perfusion to the culprit basal septum and no ethanol leakage to any other vessel (*Panel E*, *Movie S2*). Injection of 1.2 mL of ethanol resulted in ablation of the residual myocardial bulge (*Panel F*). The gradient reduced from 42 to 2 mmHg at rest and 87 to 6 mmHg on Valsalva with peak serum creatine kinase level of 453 U/L. Cardiac magnetic resonance imaging proved the additional ablation of the most-basal septum (*Panels G* and *H*, arrowheads) next to the scar of the initial ASA (*Panels G* and *I*, arrows). No relapse of LVOTO was observed for three-year follow-up.

This case illustrates that using a microcatheter for a small, tortuous septal sub-branch can be a viable alternative to an over-the-wire balloon, potentially achieving complete relief of LVOTO.

(*A*) At the initial ASA, the major septal branch originating from the left anterior descending artery was targeted (arrowhead). (*B*) Long axis and (*C*) short axis views of transthoracic echocardiography confirmed successful ablation of the targeted myocardial bulge (arrowhead). (*D*) At the repeat ASA, coronary angiography identified a sub-branch from the left circumflex artery as the target branch (arrowhead). (*E*) Tip-injection through the Corsair Pro microcatheter wedged in the sub-branch (arrowhead) confirmed perfusion to the culprit basal septum. (*F*) Intraprocedural transthoracic echocardiography showed ablation of the most-basal septum (arrowhead). (*G*) Cardiac magnetic resonance imaging revealed both acute and chronic ablation scars on a three-chamber view, with two lines indicating the planes of the short axis views presented in *Panels H* and *I*. The arrowheads in *Panels G* and *H* indicate acute microvascular obstruction at the most basal part of the septum, while the arrows in *Panels G* and *I* highlight late gadolinium enhancement, indicating the chronic scar formed by the initial ASA.

## Supplementary Material

ytae485_Supplementary_Data

## Data Availability

The data underlying this article are available in the article and in its online Supplementary material.

## Supplementary material

**Consent:** The authors confirm that written consent for submission and publication of this case report including images and associated text has been obtained from the patient in line with the Committee on Publication Ethics guidelines.

**Funding:** This study was supported by Sakakibara Heart Foundation Cardiovascular Research Program 2023 (to T.H., M.K., I.T., and M.T.).

